# Glucose concentration does not affect degradation of a protein that aberrantly engages the endoplasmic reticulum translocon

**Published:** 2020-05-08

**Authors:** Courtney L Broshar, Eric M Rubenstein

**Affiliations:** 1 Ball State University, Department of Biology, Muncie, IN 47306

## Description

**Figure 1:**
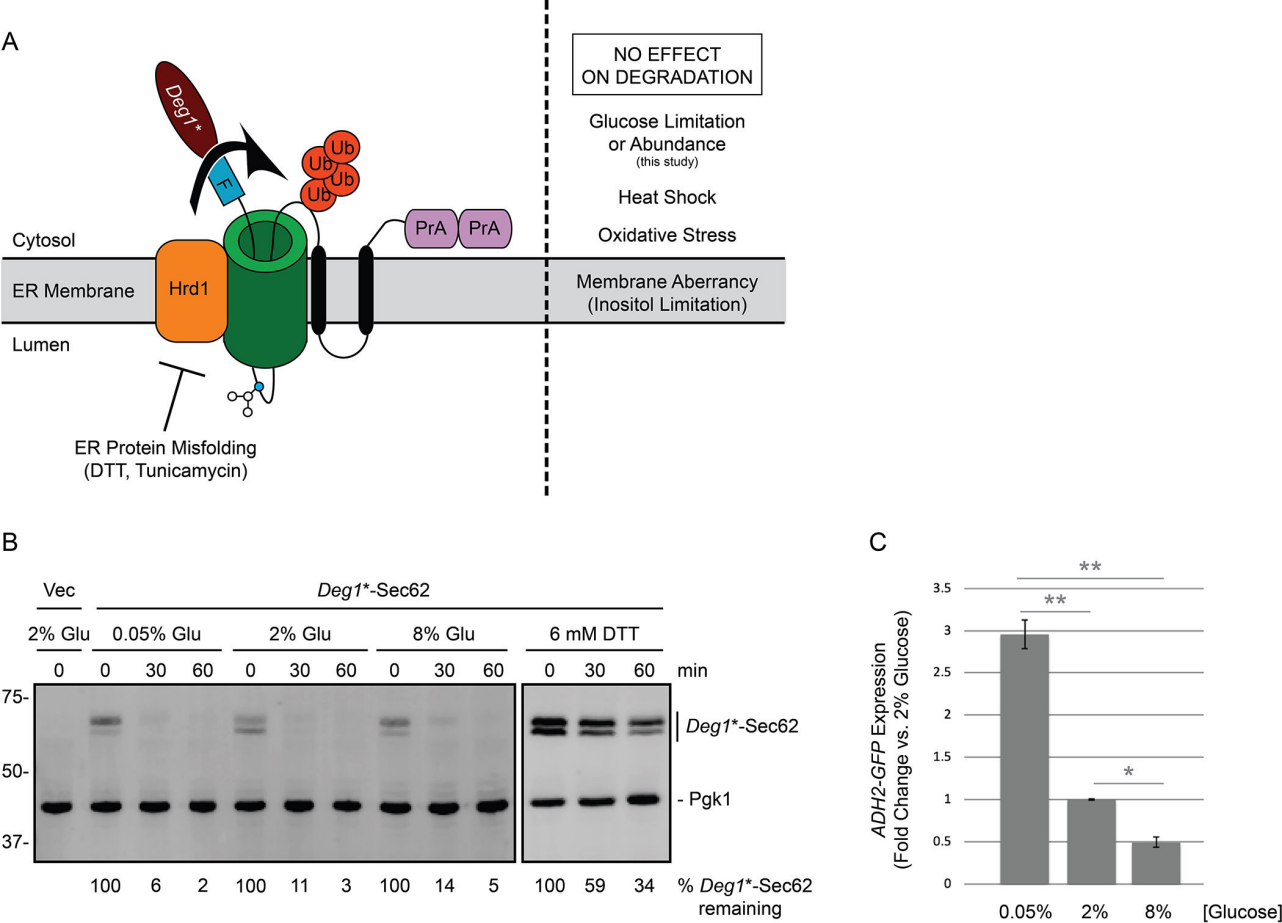
Deg1*-Sec62 degradation is unaffected by changes in glucose concentration. **(A)** Depiction of Deg1*-Sec62 following aberrant translocon engagement. Deg1*-Sec62 consists of Deg1* (a modified version of the amino-terminal 67 amino acids from the yeast transcriptional repressor MATα2p), a Flag (F) epitope, the 2-transmembrane protein Sec62p, and two copies of Protein A (PrA) from *S. aureus*. Following translocon engagement, Deg1*-Sec62 is modified by N-linked glycosylation and is targeted for degradation by the Hrd1p ubiquitin ligase (Rubenstein *et al.* 2012). Deg1*-Sec62 degradation is specifically impaired by stress caused by ER protein misfolding (Buchanan *et al.* 2019). The primary glycosylated asparagine amino acid is portrayed as a blue circle. Ub, ubiquitin. **(B)** Cycloheximide chase of yeast expressing Deg1*-Sec62 cultured in media containing 2% glucose and shifted to media containing glucose at the indicated concentrations for two hours or media containing 6 mM DTT and 2% glucose for one hour. Deg1*-Sec62 signal intensity was normalized to Pgk1p, and the percentage of Deg1*-Sec62 remaining at each time point is presented below the image. Vec, empty vector. Glu, glucose. **(C)** Parallel cultures of yeast expressing *ADH2-GFP* were cultured to mid-exponential phase growth in media containing 2% glucose and shifted to media containing glucose at the indicated concentrations for two hours before analysis by flow cytometry. The mean fluorescence intensity for each culture was normalized to the average mean fluorescence intensity of three repeats of cells incubated in the presence of 2% glucose. Mean fluorescence intensity is presented for three repeats of 10,000 cells for each condition. Error bars represent standard error of the mean. Data were analyzed by one-way ANOVA, followed by Tukey post-hoc analysis (*, *P* < 0.05; **, *P* < 0.01). Experiments depicted in this figure were performed three times.

Approximately one third of eukaryotic proteins enter the endoplasmic reticulum (ER) en route to their subcellular or extracellular destinations (Chen *et al.* 2005; Choi *et al.* 2010). Many of these proteins use the Sec61p translocon complex to cross the ER membrane (Aviram and Schuldiner 2017). Proteins that persistently engage the translocon prevent other proteins from reaching the ER (Izawa *et al.* 2012; Ast *et al.* 2016). Thus, cells have evolved multiple quality control mechanisms to degrade proteins that aberrantly occupy this channel (Rubenstein *et al.* 2012; Crowder *et al.* 2015; Ast *et al.* 2016). In ER-associated degradation of translocon-associated proteins (ERAD-T), such polypeptides are targeted for destruction by homologs of the ER-resident RING (really interesting new gene) domain ubiquitin ligase Hrd1p. Deg1*-Sec62 is an engineered model translocon-associated substrate for Hrd1p in yeast (Figure 1A). Analogously, in mammalian cells, the Hrd1p homolog gp78 promotes turnover of the low-density lipoprotein (LDL) component apolipoprotein B, which stalls in the translocon if it is unable to associate with LDL lipid molecules (Fisher *et al.* 2011).

We recently discovered that degradation of Deg1*-Sec62 is impaired by ER stress (the accumulation of misfolded or unfolded proteins in the ER). Deg1*-Sec62 is strongly stabilized by treatment with dithiothreitol (DTT; which reduces disulfide bonds) or tunicamycin (which prevents N-linked glycosylation). By contrast, however, Deg1*-Sec62 degradation is unaffected by perturbation of ER membrane lipid composition (i.e. inositol limitation) or treatments expected to broadly perturb proteostasis (elevated temperature or oxidative stress) (Buchanan *et al.* 2019).

The AMP-activated protein kinase Snf1p is stimulated during ER stress (Mizuno *et al.* 2015). Further, loss of the Snf1p inhibitor Reg1p renders cells hypersensitive to ER stress (Ferrer-Dalmau *et al.* 2015). Snf1p is also regulated by nutrient abundance; it is activated by phosphorylation when glucose is limiting and inactivated by dephosphorylation when glucose is abundant (Rubenstein *et al.* 2008). Given ERAD-T sensitivity to ER stress and crosstalk between ER stress and nutrient stress signaling, we sought to determine if turnover of the ERAD-T substrate Deg1*-Sec62 is regulated by changes in glucose abundance.

We performed cycloheximide chase experiments to compare Deg1*-Sec62 degradation kinetics in low (0.05%), standard (2%), or high (8%) glucose concentrations (Figure 1B). Deg1*-Sec62 was rapidly degraded in all three conditions. By contrast, DTT strongly stabilized and impaired post-translational modification of Deg1*-Sec62, as previously reported (Buchanan *et al.* 2019). *ADH2* expression is repressed by glucose (Dombek *et al.* 1993). To confirm differences in glucose abundance, *ADH2-GFP* expression was compared using flow cytometry of a parallel culture (Figure 1C). Our results indicate that changes in glucose abundance (in the range of 0.05% to 8%) do not substantially alter the rate of degradation of Deg1*-Sec62, a model translocon-associated substrate of Hrd1p.

Taken with our recently published work (Buchanan *et al.* 2019), our results indicate that ERAD-T is inhibited by stress caused by ER protein misfolding but not membrane stress, oxidative stress, heat shock, or glucose limitation or abundance. It remains possible that altered glucose levels exert an effect on ERAD-T in the context of ER stress or mutations in genes mediating crosstalk between ER stress and nutrient signaling. Future experiments may be performed to test these hypotheses. During ER stress, protein translocation into the ER is slowed (Kang *et al.* 2006). We speculate that inhibited degradation of proteins that persistently engage the translocon contributes to reduced overall rates of translocation, preventing an already stressed ER from becoming overwhelmed.

## Methods

### Yeast and Plasmid Methods

Yeast were cultured at 30°C in synthetic-defined growth media (Guthrie and Fink 2004). An empty vector (pVJ27/pRS316; *URA3*/CEN (Sikorski and Hieter 1989)) and a plasmid encoding Deg1*-Sec62 driven by the *MET25* promoter (pVJ317; *URA3*/CEN (Rubenstein *et al.* 2012)) were introduced to yeast (VJY476/BY4741 *MATa his3*Δ*1 leu2*Δ*0 met15*Δ*0 ura3*Δ*0* (Tong *et al.* 2001)) via lithium acetate transformation (Guthrie and Fink 2004). Yeast expressing *ADH2* with a C-terminal GFP tag (VJY731; *MATa his3*Δ*1 leu2*Δ*0 met15*Δ*0 ura3*Δ*0 ADH2-GFP:HIS3MX6*) were obtained from the Yeast GFP Clone Collection (Invitrogen (Huh *et al.* 2003)).

### Flow Cytometry

Yeast expressing *ADH2-GFP* were cultured, in triplicate, to mid-exponential growth at 30°C in media containing 2% glucose, washed five times in media containing 0.05%, 2%, or 8% glucose, and incubated in fresh media containing the same glucose concentrations for two hours, as indicated. Mean GFP fluorescence of 10,000 cells was measured using the MACSquant Analyzer X.

### Cycloheximide Chase Analysis, Cell Lysis, and Western Blotting

Cycloheximide chase analysis was performed as described previously (Buchanan *et al.* 2016). For glucose treatments, yeast cultured to mid-exponential phase growth in media containing 2% glucose were washed five times in media containing 0.05%, 2%, or 8% glucose and incubated in fresh media containing the same glucose concentrations for two hours at 30°C. For cultures treated with dithiothreitol (DTT), DTT was added to mid-exponential phase cultures (6 mM DTT final concentration) for one hour of incubation at 30°C. Glucose and DTT concentrations were maintained throughout the course of the cycloheximide chase. Proteins were extracted and analyzed by western blotting as described previously (Kushnirov 2000; Watts *et al.* 2015). Deg1*-Sec62 is C-terminally tagged with two copies of the *Staphylococcus aureus* protein A epitope (Figure 1A). *S. aureus* Protein A binds to mammalian immunoglobulins (Hjelm *et al.* 1972); therefore, AlexaFluor-680-conjugated rabbit anti-mouse antibody (Life Technologies, Inc; 1:40,000) was used to directly detect Deg1*-Sec62. Pgk1p was detected with mouse anti-phosphoglycerate kinase 1 (Pgk1; clone 22C5D8; Life Technologies, Inc; 1:20,000) followed by AlexaFluor-680-conjugated rabbit anti-mouse secondary antibody (1:40,000). Membranes were imaged and analyzed using an Odyssey CLx Infrared Imaging System and Image Studio Software (Li-Cor).
